# Effects of intervalley scattering on the transport properties in one−dimensional valleytronic devices

**DOI:** 10.1038/srep23211

**Published:** 2016-03-16

**Authors:** Jiaojiao Zhou, Shuguang Cheng, Wen-Long You, Hua Jiang

**Affiliations:** 1 College of Physics, Optoelectronics and Energy, Soochow University, Suzhou, 215006, China; 2 Department of Physics, Northwest University, Xian 710069, China

## Abstract

Based on a one-dimensional valley junction model, the effects of intervalley scattering on the valley transport properties are studied. We analytically investigate the valley transport phenomena in three typical junctions with both intervalley and intravalley scattering included. For the tunneling between two gapless valley materials, different from conventional Klein tunneling theory, the transmission probability of the carrier is less than 100% while the pure valley polarization feature still holds. If the junction is composed of at least one gapped valley material, the valley polarization of the carrier is generally imperfect during the tunneling process. Interestingly, in such circumstance, we discover a resonance of valley polarization that can be tuned by the junction potential. The extension of our results to realistic valley materials are also discussed.

Valley is a new degree of freedom of the charge carriers. It exists in a crystalline material with energetically degenerate but inequivalent band structures. In recent years, the community have generated extensive interest in manipulating and controlling of the valleys^1^,^2^,^3^, and thus stimulate a blooming field of valleytronics. A number of materials, including graphene^4^, layered transition metal dichalcogenides^5^ and Weyl semimetal system^6^ etc, are well known hosts of the valleys. Based on them, there are plenty of researches on valleytronics on both theoretical^1^,^2^,^7^,^8^,^9^,^10^,^11^,^12^,^13^,^14^,^15^,^16^,^17^,^18^,^19^,^20^,^21^,^22^,^23^,^24^ and experimental sides^24^,^25^,^26^,^27^,^28^,^29^,^30^,^31^,^32^,^33^ in the past few years. To be specific, following the theoretical prediction^2^,^7^,^24^, several groups have successfully created valley polarization and detected its signal through the optical pumping by circularly polarized photons^24^,^25^,^26^ and the valley Hall effect^28^,^29^,^30^,^31^ in various valley materials, which made a great progress on the valleytronics.

Subsequently, manipulating the transport of valley polarized carriers becomes an important issue in the velleytronics research. Different from the charge and spin degrees of freedom, there is no symmetry to guarantee the conservation of the valleys. In a realistic material, any potential difference, such as disorder, interface mismatch, surface effect and boundary roughness ect, can induce scattering between different valleys, which may lead to the mixture of the valleys^22^. Transport experiments demonstrate that there is a characteristic length, similar to localization length and phase relaxation length, namely intervalley scattering length, determines the valley-related phenomena. The valley-dependent effects decay exponentially with such length scale^28^,^29^,^30^,^31^,^32^,^33^. Thus, reducing intervalley scattering is crucial for highly efficient valleytronic devices. However, in most of previous theoretical studies, the intervalley scattering is neglected by concentrating the studies on a single valley or assuming that the potential is smooth enough^1^,^2^,^7^,^10^,^11^,^12^,^13^,^20^,^21^,^23^,^24^. In this regard, an immediate question is to what extent could the valley transport properties be affected by intervalley scattering in valleytronic devices.

In this paper, we address such an issue on the basis of investigating the valley transport properties in one-dimensional valley material junction devices. By solving the model analytically, the influences of the both intervalley and intravalley scattering on the junctions between two gapless, one gapless and one gapped, and two gapped valley materials are obtained. Due to the intervalley scattering, the Klein tunneling theory, which predicts that the carriers experience a perfect tunneling with 100% transmission probability between two gapless valley materials, is invalid^34^,^35^. However, the pure valley polarization feature of the transmitted carriers still holds. If the junctions contain gapped valley materials at least in one terminal, the intervalley scattering always lead to a partial change of the valley polarization during the tunneling process. Under this circumstance, the valley polarization ratio can be manipulated by tuning the junction potential due to the existence of the valley polarization resonance phenomena. Finally, we discuss the extension of our findings to two- and three-dimensional valley materials.

## Results

### Model

**Figure 1:**
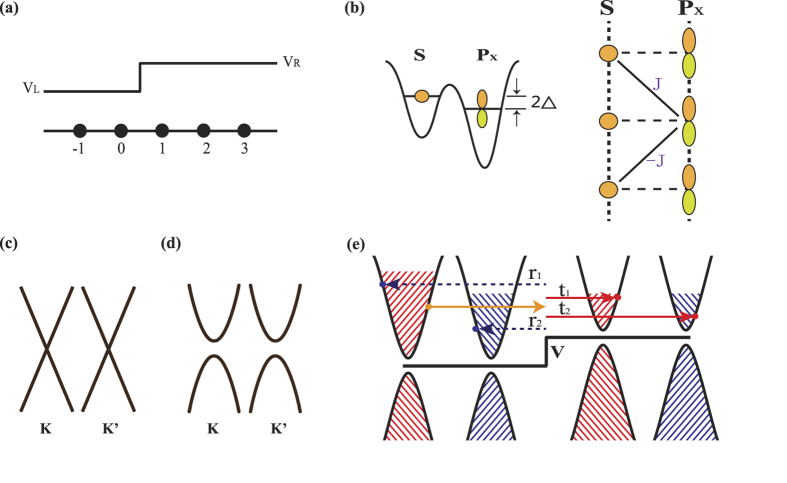
Illustration of studied model. (**a**) Schematic diagram of one-dimensional valley junction device, *V*_*L*_, *V*_*R*_ are the site potential on the left and right side; (**b**) illustrates a proposal of orbit and lattice geometries for the realization of our ideal valley model; (**c**,**d**) show valley configurations of the model under Δ = 0 (c) and Δ ≠ 0 (**d**). (**e**) Schematic plot of the a valley tunneling process: an incident valley polarized mode will have two transmitted valley modes with the tunneling coefficients *t*_1_ and *t*_2_ and two reflected valley modes with the reflection coefficients *r*_1_ and *r*_2_.

To study the valley transport, we begin with constructing a minimal lattice model. As sketched in Fig. 1(a,b), we consider a junction device with one-dimensional valley materials on both sides. In the tight-binding representation, the effective Hamiltonian can be written as:


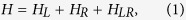


in which


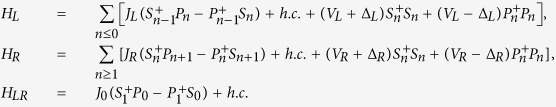


Here *H*_*L*_, *H*_*R*_ and *H*_*LR*_ describe the left side, the right side and the tunneling coupling in between, respectively. There are two orbits *S*, *P*_*x*_ for each site *n* and *S*_*n*_, *P*_*n*_ denote the corresponding annihilation operators. *V*_*L*_(*V*_*R*_) characterizes the on-site potential and Δ_*L*_(Δ_*R*_) specifies the site potential difference of these two orbits on left (right) side. We assume that the electron hopping can only happen between the *S* orbit and the nearest-neighbor site *P*_*x*_ orbits [see Fig. 1]. *J*_*L*_, *J*_*R*_ and *J*_0_ are the hopping energies on the left side, the right side and the junction interface, respectively. We note that our minimal model can be realized in the special ultracold atom systems, where every parameter in the Hamiltonian can be tuned physically (please see ref. 36 for details). In the following, we set *J*_*L*_ = *J*_*R*_ = 1, *V*_*L*_ = 0 and *V*_*R*_ = *V* for convenience.

We perform a Fourier transformation to Eq. (1) and obtain its form in the momentum space:


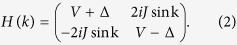


For simplicity, we have set the lattice constant *a* = 1. *H*(*k*) has two eigenvalues 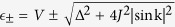, where 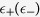 corresponds to the conduction (valence) band. Figure 1(c) plots the energy dispersion of *H*(k) for Δ = 0 and Δ ≠ 0. The energy band are degenerate at k = 0 (*K*) and k = *π* (*K*′), which means that there exist two inequivalent valley *K* and *K*′. Moreover, two types of valleys can be realized by tunneling Δ. One type is gapless as illustrated in Fig. 1(c) and the other has gapped spectrum in Fig. 1(d). In a word, the minimal model provides a platform to study the valley transport properties between the valley materials, in both gapless and gapped situations.

In the following, we focus on the valley transport properties for the junction devices. The incident carriers are purely *K*-valley polarized. The valley-resolved transmission probabilities *T*_*KK*_, *T*_*KK*′_, reflection probabilities *R*_*KK*_, *R*_*KK*′_, the total transmission probability *T* and the valley polarization ratio *P* are obtained analytically. See **Methods** for details of these calculations.

In recent years, lots of gapless or gapped valley materials have been discovered. In general, three types of valley junction can be built through these materials: gapless junction between two gapless valley materials (Δ_*L*_ = Δ_*R*_ = 0); hybridized junction with gapless and gapped valley materials (Δ_*L*_ = 0, Δ_*R*_ ≠ 0 or Δ_*L*_ ≠ 0, Δ_*R*_ = 0); gapped junction between two gapped valley materials (Δ_*L*_ ≠ 0, Δ_*R*_ ≠ 0). Without loss of generality, we consider these three cases separately.


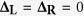


In this subsection, we study the transport properties of a junction between two gapless valley materials. The considered situation is analogue to the valley transport properties in graphene, silicence, and germanene PN junction etc^37^,^38^. Conventionally, the Klein tunneling theory is used to describe the transport phenomena in the gapless material systems^34^,^35^,^39^. In such theory, only single valley is considered. Thus, due to the absence of intervalley scattering, the valley polarization will never be changed during the scattering processes (*P* = 1). Moreover, the gapless feature forbids the intravalley backscattering (*r* = 0, see the second part of section **Methods**). The carrier can tunnel through the barrier freely (*T* = 1). However, in real materials, the *fermion doubling theorem* guarantees that the gapless valleys exist in pairs^40^. Thus, the intervalley scattering is unavoidable due to the interfacial complexity.

**Figure 2:**
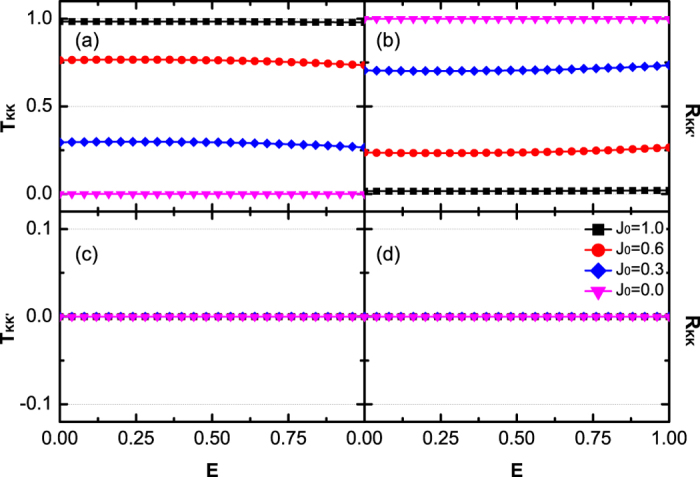
Valley-resolved transmission probabilities **T**_**KK**_ (**a**), **T**_**KK′**_ (**c**) and reflection probabilities **R**_**KK′**_ (**b**), **R**_**KK**_ (**d**) versus Fermi energy **E** for different interfacial hopping energy **J**_**0**_. The interfacial potential of the junction is fixed to *V* = 0.5. The hopping energies on both sides of the junction are *J*_*L*_ = *J*_*R*_ = 1.

To show an intuitive picture for the influence of intervalley scattering, we firstly study the junction in an extreme condition that large mismatch exists at the interface, that is, the hopping energy *J*_0_ is much different from *J*_*L*_, *J*_*R*_. Figure 2 plots the valley resolved probability *T*_*KK*_ (a), *R*_*KK*′_ (b), *T*_*KK*′_ (c) and *R*_*KK*_ (d) versus the Fermi energy *E* for different hopping energy *J*_0_. At first glance, *T*_*KK*′_ and *R*_*KK*_ are equal to zero, independent with *J*_0_ and *E*. *T*_*KK*′_ = 0 means that the *K* valley polarized carriers on left side cannot tunnel into the *K*′ valley on the right side. Thus, the valley polarization (*P* = 1) will not be changed during the tunneling processes. *R*_*KK*_ = 0 implies that the *K* valley carriers cannot be reflected back to its own valley. These two features agree well with the Klein theory. But, in such gapless system, the total transmission probability (*T* = *T*_*KK*_ = 1) predicted in Klein theory is violated. As shown in Fig. 2(a), *T*_*KK*_ is not equal to 1. By decreasing *J*_0_, *T* = *T*_*KK*_ drop quickly. This phenomenon stems from the fact that the intervalley scattering is allowed for the existence of pairs of valleys. As seen from Fig. 2(b), the *K* valley carrier can be reflected to the *K*′ valley. A decrease of *J*_0_ leads to a rapid increase of *R*_*KK*′_.

*J*_0_ is not the only mechanism that can cause the intervalley scattering. Even though *J*_0_ = *J*_*L*_ = *J*_*R*_, intervalley scattering still emerges by tuning the junction potential *V*. In the following, we will focus on this case, because it is much closer to the experimental situation. We find that the influence of V for a single junction is weak. However, in realistic experimental situations, the carriers may experience a series of junctions during the tunneling process, thus the influence of V will be enhanced.

**Figure 3:**
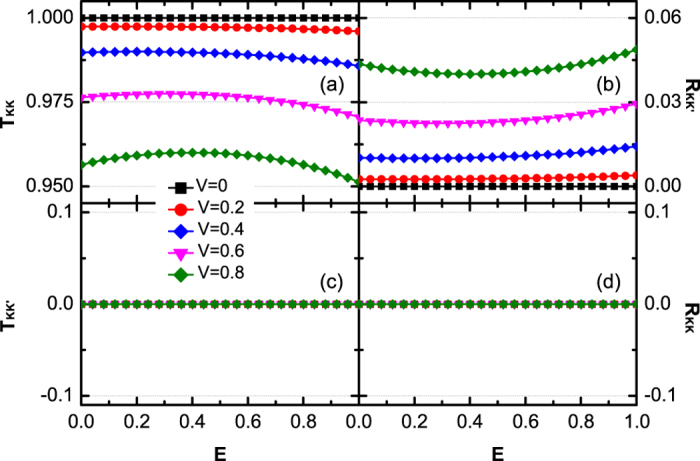
Transmission probabilities **T**_**KK**_ (**a**), **T**_**KK′**_ (**c**) and reflection probabilities **R**_**KK′**_ (**b**), **R**_**KK**_ (**d**) vs Fermi energy E for different potential **V = 0.0, 0.2, 0.4, 0.6, 0.8**. All hopping energies are set to be equal, *J*_0_ = *J*_*L*_ = *J*_*R*_ = 1.

**Figure 4:**
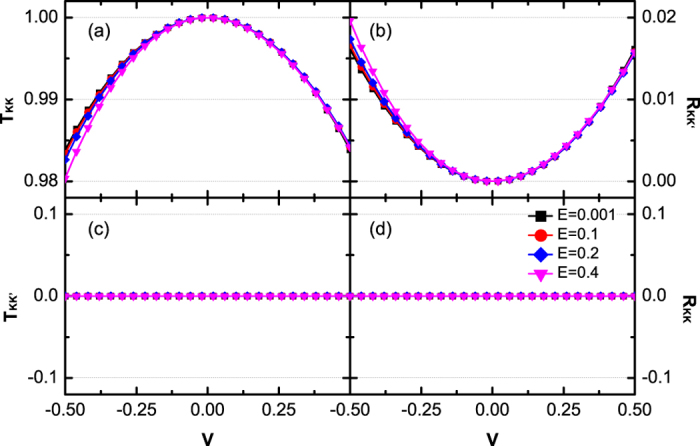
Transmission probabilities **T**_**KK**_ (**a**), **T**_**KK′**_ (**c**) and reflection probabilities **R**_**KK′**_ (**b**), **R**_**KK**_ (**d**) versus potential **V** for different Fermi energy **E = 0.0001, 0.1, 0.2, 0.4**. The hopping energies are the same as Fig. 3.

Figures 3 and 4 show the relationship about transmission probabilities *T*_*KK*_(a), *T*_*KK*′_(c) and reflection probabilities *R*_*KK*′_(b), *R*_*KK*_ (d) with the Fermi energy *E* and the potential *V*. In Figs 3(c,d) and 4(c,d), *T*_*KK*′_ and *R*_*KK*_ still equal to zero, as same as those in Fig. 2(c,d). This phenomenon indicates that the valley polarization cannot be changed (*T*_*KK*′_ = 0 and *P* = 1) during the tunneling processes. By changing the potential *V* or the Fermi energy *E*, the probability that the *K*-polarized carriers tunnel into the valley *K* (*T*_*KK*_ ≠ 1) or been reflected into the valley *K*′ (*R*_*KK*′_ ≠ 0) can be adjusted. As plotted in Fig. 3, *T*_*KK*_ has an obvious decrease and *R*_*KK*′_ shows an obvious increase with respect to large *V*. In contrast, in Fig. 4, *T*_*KK*_ and *R*_*KK*′_ change slowly by varying the Fermi energy *E*. These two behaviors can be understood by the analytic expression of *R*_*KK*′_ under the situation Δ_*L*_ = Δ_*R*_ = 0. From Eqs (9) and (10), one obtains


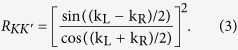


When Fermi level *E* is near the Dirac point, both k_*L*_ and k_*R*_ are very small. The junction keeps a linear dispersion on both sides. As a consequence, k_L_ − k_R_ is proportional to the potential *V* and *cos*(k_L_ + k_R_) is insensitive to the variation of Fermi energy *E*. Therefore, *T*_*KK*_ and *R*_*KK*′_ are sensitive to the potential *V* due to the rapid variation of k_L_ − k_R_. When *V* is fixed and the energy structure is in linear dispersion regime, *T*_*KK*_ and *R*_*KK*′_ change slowly with *E* due to small variation of cos(k_L_ + k_R_). When *E* is shifted away from the linear dispersion regime, the total transmission probability *T* = *T*_*KK*_ deviates significantly from unit.


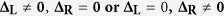


In this subsection, we study transport properties of a junction in which only one side has gapped valleys. In this circumstance, valley-polarized carriers are injected from gapless valley materials to gapped valley materials or vice verse. This hybrid junction can be realized in experiments. For example, we can design a junction composing of a monolayer MoS_2_ hybridized with graphene sheet or a junction for bilayer graphene with unbiased left side and biased right side^41^.

**Figure 5:**
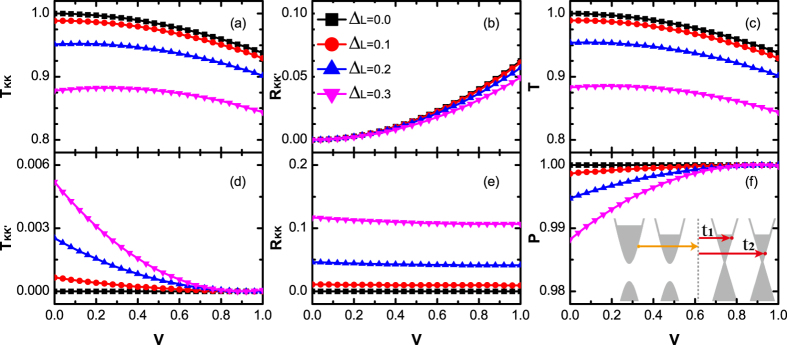
The valley resolved transmission probabilities **T**_**KK**_ (**a**), **T**_**KK′**_ (**d**), total transmission probabilities **T** (**c**), reflection probabilities **R**_**KK′**_ (**b**), **R**_**KK**_ (**e**) and valley polarization ratio **P** (**f**) versus interfacial potential V. The valley gap on the left side equal to 0.0, 0.1, 0.2, 0.3, respectively. The other parameters are set as: the valley gap on the right side Δ_*R*_ = 0, the Fermi energy *E* = 0.5 and the hopping energy *J*_0_ = *J*_*L*_ = *J*_*R*_ = 1. Inset of (**f**) is the schematic diagram for the carrier tunneling process.

**Figure 6:**
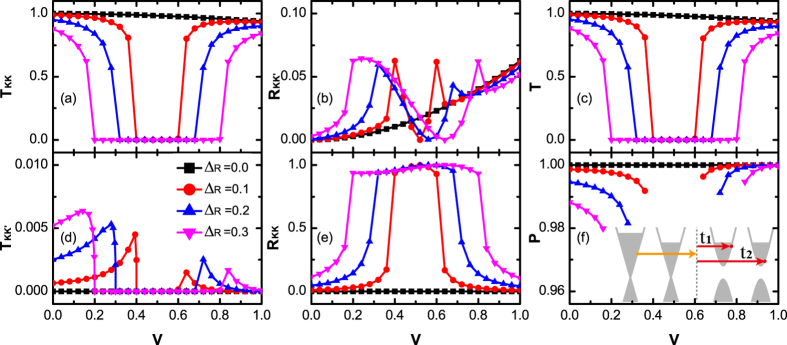
The valley resolved transmission probabilities **T**_**KK**_ (**a**), **T**_**KK′**_ (**d**), the transmission probabilities *T* (**c**), reflection probabilities **R**_**KK′**_ (**b**), **R**_**KK**_ (**e**) and the valley polarization ratio **P** (**f**) versus interfacial potential **V**. The valley gap is on the right side, Δ_*L*_ = 0, Δ_*R*_ ≠ 0. The Fermi energy *E* and the hopping energies *J*_0_, *J*_*L*_, *J*_*R*_ are the same as those in Fig. 5. Inset of (**f**): schematic of the tunneling process for valley polarized carriers.

In Figs 5 and 6, *T*_*KK*_, *T*_*KK*′_, *R*_*KK*_, *R*_*KK*′_ as a function of potential *V* for gapped valleys on left-side (Fig. 5) or right-side (Fig. 6) of junction are plotted, respectively. The corresponding probabilities of the gapless junction studied in the above subsection (Δ_*L*_ = Δ_*R*_ = 0) are also plotted in these two figures (black square curves) for comparison. Remarkably, when either Δ_*L*_ ≠ 0 or Δ_*R*_ ≠ 0, both *T*_*KK*′_ and *R*_*KK*_ are always nonvanishing. Owing to the existence of the additional intravalley backscattering, the transmission probabilities *T* is weakened. More importantly, with the occurence of the intervalley transmission, the valley polarization ratio *P* is less than the unit. In the following, we investigate how *T* and *P* are affected in detail.

In Fig. 5(c), the transmission probabilities *T* under different valley gap Δ_*L*_ and potential *V* are plotted. *T* decrease rapidly by increasing the gap Δ_*L*_ and the potential *V*. The variation of *T* versus Δ_*L*_ is mainly contributed by the dominant reduction of *T*_*KK*_ for larger Δ_*L*_. It can be understood by the transport theory with single valley (see section **Methods** for detail). Meanwhile, the variation of *T* versus *V* follows its tendency at Δ_*L*_ = 0 (black line). The phenomena originates from the same mechanism that a rise of *V* will sharply enhance the intervalley scattering *R*_*KK*′_ (see Fig. 5(b)). Furthermore, the relationship of valley-polarization ratio *P* versus potential *V* for different Δ_*L*_ are shown in Fig. 5(f). First, in the present model, no perfect valley polarization can be realized as long as Δ_*L*_ > 0. Second, *P* is proportional to *V* while in inverse proportion to Δ_*L*_. Such relationship is determined by the variation of *T*_*KK*′_ versus *V* and *T*. For fixed potential *V*, the band structure on the both sides of the junction becomes more asymmetrical by increasing of Δ_*L*_, leading to an enhancement of *T*_*KK*′_. In contrast, by increasing *V*, *T*_*KK*′_ decreases since the band structure on the both sides of the junction becomes more symmetrical. From above relationship, one can conclude that the polarization ratio *P* can be manipulated by controlling *V*. To be specific, for fixed Δ_*L*_, the valley-polarization *P* can be greatly improved with little expense of *T* by increasing *V*.

In Fig. 6(c,f), we investigate the case that nonzero valley gaps only emerge on the right side of the junction. Comparing with the curves that Fermi energy shifts from the valence band to the conduction band, the behaviors of *T* and *P* are nearly the same. In other words, both *T* and *P* are insensitive to the type of carriers (p/n). Further, when Fermi energy *E* approaches the band gap, both transmission probabilities *T* and valley polarization ratio *P* decrease rapidly. From the relationship of *P* versus Δ_*L*_ and *V* (see Fig. 6(f)), it is also worth emphasizing that, due to the intervalley scattering, the valley polarization ratio *P* is generally less than unit. Meanwhile, one can also manipulate *P* by adjusting the potential *V*.


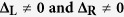


After the discovery of series of gapped valley materials recently^3^,^5^, great interest has been sparked in the study of valley transport in these materials. In this subsection, we study the transport properties of a junction between two gapped valley materials.

**Figure 7:**
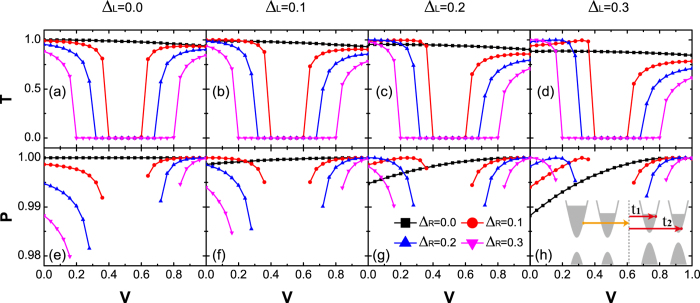
The total transmission probability T (**a–d**), and the valley polarization ratio P (**e–h**) as functions of V and the valley gap on the right side **Δ**_**R**_. (**a,e**), (**b,f**), (**c,g**) and (**d,h**) correspond to Δ_*L*_ = 0.0, 0.1, 0.2, 0.3. The other parameters are the same as those in Fig. 5. Inset of (**h**) illustrates the valley tunneling process.

Figure 7 plots the transmission probabilities *T* and the valley polarization ratio *P* versus potential *V* for different band gaps. Main features of relationship between *T*, *P* and *V*, Δ are obtained. In general, due to the intervalley scattering, *P* is less than 1, indicating that partial carriers will change their valley; *T* is not identical to 1, which means that the backscattering takes place at the interface; *T* and *P* decrease rapidly at the gap edges.

Interestingly, in the presence of gapped valley materials, resonance phenomena happen to *P* (see Figs 5 and 7). In other words, at some special values of *V* (e.g., *V* = 0.167 or *V* = 0.947 in Fig. 7(h)), the valley polarization will not be changed during the tunneling process (*P* = 1). We have carefully investigated such resonance phenomenon since it may have potential application in the manipulation of valley transport.

**Figure 8:**
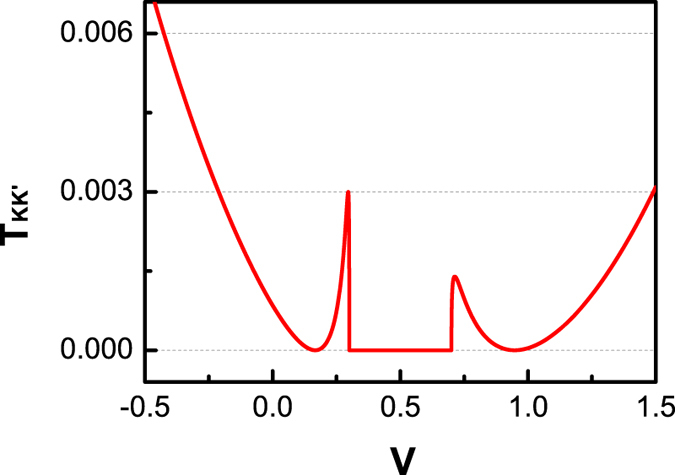
The valley-resolved probability **T**_**KK′**_ versus potential V in the case of **Δ**_**L**_** = 0.3** and **Δ**_**R**_** = 0.2**. The other parameters are the same as those in Fig. 5.

Physically, the *P* resonance is caused by the vanishment of intervalley transmission probability *T*_*KK*′_. Figure 8 illustrates *T*_*KK*′_ as a function of *V*. One can see two zero points for *T*_*KK*′_. After some algebras, we find that the resonance points locate at


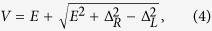


or


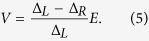


Noting the first type valley resonance phenomenon [Eq. (4)] can also exist in the hybridized junction that only Δ_*L*_ = 0 or Δ_*R*_ = 0. In contrast, the second type valley resonance phenomenon [Eq. (5)] can only exist in the junction with gapped valleys on both sides (Δ_*L*_ ≠ 0 and Δ_*R*_ ≠ 0). To be specific, when Δ_*L*_ approaches Δ_*R*_, a pure valley polarization can easily be obtained by slightly tuning the potential *V*. Equation (5) is the master equation that determines such valley manipulation.

## Discussion and Conclusion

Commonly, the valley materials that have been successfully fabricated in experiments are two- or three-dimensional with complicated electric structure^42^,^43^,^44^,^45^. In the main text, we concentrate our studies on a simple one-dimensional valley junction model. However, the underlying physical mechanism of the valley transport properties even in the complicated materials has been clearly demonstrated in such a simple model. Taking a junction composed of two-dimensional gapless valley materials (e.g. monolayer graphene) for example, its momentum can be decoupled into the perpendicular component k_⊥_ and the parallel component k_||_ along the junction interface. The eigenvalues and eigenstates are similar to those in Eq. (2) except that Δ is replaced by the momentum k_||_. Due to the continuity relationship of the wavefunction, Eq. (9) still holds and k_||_ remains unchanged across the junction. Therefore, for normally incident carriers, their valley transport should show similar behaviors as elaborated in subsection **Δ**_**L**_** = Δ**_**R**_** = 0**. For carriers with an incident angle, their valley transport properties should share similar features to those observed in subsection **Δ**_**L**_ ≠ **0** and **Δ**_**R**_ ≠ **0** with k_||_ = Δ_*L*_ = Δ_*R*_. Parallel analysis can be applied to the two-dimensional gapped valley materials and three-dimensional valley materials, and their valley transport properties will resemble the last three subsections in Results, depending on the initial condition. Conclusively, our analytic results can also characterize the main features of valley transport phenomena in both two- and three-dimensional valley materials.

In summary, the valley transport properties of a junction between one-dimensional materials with gapless (or gapped) valleys are studied. Our analytic results have clearly shown that the strong intervalley scattering, which is always omitted in the previous theory, can greatly influence the valley transport properties of valleytronic devices. Concretely, for a junction with two gapless valley materials, the intervalley scattering can cause the reflection of the tunneling carriers and damage the perfect tunneling. Nevertheless, the valley polarization of the carriers remians unchanged in such a case. In contrast, for a junction containing gapped valley materials, the valley polarization of the carriers can be changed during the tunneling process. Besides, we discover a valley polarization resonance phenomena and extract the corresponding condition, which may be utilized to manipulate the valley degree in the future.

## Methods

### Valley transport with intervalley scattering included

As shown in Fig. 1(e), for the incident mode Φ_*in*_, there are two reflection modes Φ_*r*1_, Φ_*r*2_ on the left side and two transmission modes Φ_*t*1_, Φ_*t*2_ on the right side. Φ_*in*_, Φ_*r*1_, Φ_*r*2_, Φ_*t*1_, Φ_*t*2_ on site *n* are given by:


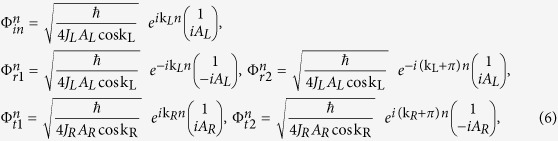


where k_L_(k_R_) represents the wavevector of the mode Φ_*in*_(Φ_*t*1_) at Fermi energy *E*. The components 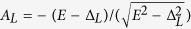 and 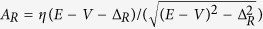. Here *η* = −1(1) corresponds to the case that *E* is located in the conduction band or the band gap (the valence band). *A*_*R*_ is purely imaginary when *E* is located inside the band gap. The two-component spinors in Eq. (6) originate from the eigenvector of *H*(*k*). It is worth noting that, in order to satisfy the unitary properties of scattering matrix, all modes in Eq. (6) have been normalized by the current operator *j* = ∂*H*(*k*)/∂*k*.

The wave function Ψ_*n*_ at site *n* can be written as a combination of the incident modes and reflected/transimitted modes in the following equations:


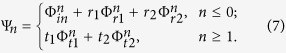


From the stationary Schrödinger equation, *H*Ψ = *E*Ψ, the continuity relationship across the interface can be expressed as:


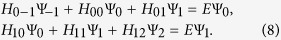


Combining Eqs (6) and (8), we have


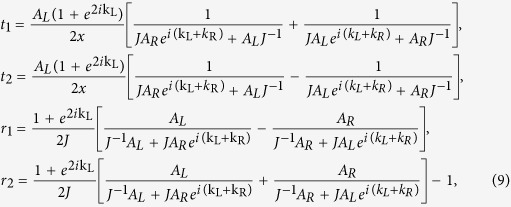


where 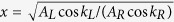 and 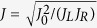. Consequently, we can get the corresponding transmission and reflection probabilities:


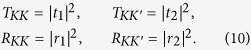


*T*_*KK*_ (*T*_*KK*′_) denotes the transmission probability of carriers from *K* valley on the left side to the *K*(*K*′) valley on the right side, and *R*_*KK*_(*R*_*KK*′_) represents the reflection probabilities of carriers from *K* valley to *K*(*K*′) valley. It could be proved that *T*_*KK*_ + *T*_*KK*′_ + *R*_*KK*_ + *R*_*KK*′_ = 1. Such constraint is guaranteed by the unitary properties of the scattering matrices and shows strong confirmation of our analytical derivations.

Based on the valley-resolved transmission probability, we can define the total transmission probability


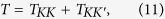


and the valley polarization ratio


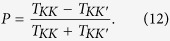


*T* and *P* are two important quantities characterize the valley transport properties of the device.

### Single valley transport theory

For single valley model, the low-energy effective Hamiltonian can be written as:


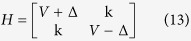


The above Hamiltonian have eigenvalues 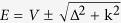. For simplicity, we focus on conduction band 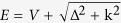 and its eigenfunction is 
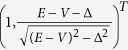
.

We consider a junction with gapless left side (Δ_*L*_ = 0) and gapped right side (Δ_*R*_ ≠ 0). The continuity of wavefunction across the boundary can be written as:


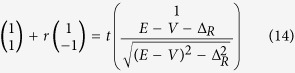


Finally, we obtain the reflection amplitude


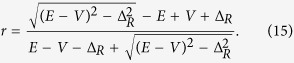


When Δ_*R*_ = 0, *r* = 0 for arbitrary *V*. It corresponds to the total transmission, which is in agreement with Klein theory for massless fermion system. In contrast, when Δ_*R*_ → *E* − *V*, the reflection amplitude *r* → 1. It corresponds to the perfect reflection.

## Additional Information

**How to cite this article**: Zhou, J. *et al.* Effects of intervalley scattering on the transport properties in onedimensional valleytronic devices. *Sci. Rep.* **6**, 23211; doi: 10.1038/srep23211 (2016).
